# Epigenetic Mechanisms in Perioperative Medicine: From Neuroinflammation and NETosis to Organ Dysfunction and Precision Therapeutics

**DOI:** 10.3390/biomedicines14081658

**Published:** 2026-07-23

**Authors:** Katharina Rump, Michael Adamzik

**Affiliations:** Ruhr University Bochum, Knappschaft Kliniken University Hospital Bochum, Department of Anesthesiology, Intensive Care Medicine and Pain Therapy, 44801 Bochum, Germany; michael.adamzik@knappschaft-kliniken.de

**Keywords:** perioperative medicine, epigenetics, perioperative neurocognitive disorders, neuroinflammation, NETosis, histone deacetylases, DNA methylation, m6A RNA methylation, fibrosis, precision medicine

## Abstract

Perioperative stress induces profound molecular and cellular responses that contribute to postoperative complications, including perioperative neurocognitive disorders (PND), chronic postsurgical pain, organ dysfunction, immunothrombosis, fibrosis, and cancer progression. Increasing evidence demonstrates that epigenetic mechanisms act as central regulators linking surgical trauma, inflammation, metabolic stress, ischemia–reperfusion injury, and immune activation to long-term alterations in gene expression and tissue remodeling. DNA methylation, histone modifications, chromatin remodeling, non-coding RNAs, and RNA epitranscriptomic mechanisms such as N6-methyladenosine (m6A) collectively orchestrate perioperative responses across multiple organ systems. Recent translational studies have identified histone deacetylases (HDACs), histone methyltransferases, NETosis-associated chromatin signaling, HMGB1/NF-κB activation, and epigenetic regulation of neuroimmune pathways as major contributors to postoperative cognitive dysfunction, chronic pain, cardiac dysfunction, pulmonary injury, and fibrosis. In parallel, advances in liquid biopsy, circulating tumor DNA (ctDNA), and single-cell epigenomics have opened new opportunities for biomarker-guided perioperative precision medicine. This review summarizes current evidence regarding epigenetic regulation in perioperative medicine with special emphasis on neuroepigenetics, NETosis, fibrosis, cardiac epigenetics, immune remodeling, and perioperative oncological outcomes. Furthermore, we discuss emerging therapeutic strategies targeting HDACs, DNA methylation, m6A pathways, and chromatin-associated inflammatory signaling as potential future interventions for perioperative complications.

## 1. Introduction

Surgery and anesthesia induce a complex systemic stress response characterized by inflammatory activation, neuroimmune signaling, endocrine dysregulation, oxidative stress, tissue injury, and metabolic remodeling [1]. Although these responses are initially adaptive, excessive or persistent activation may contribute to postoperative complications including perioperative neurocognitive disorders (PNDs) such as postoperative delirium (POD) and postoperative cognitive dysfunction (POCD) [2], chronic postsurgical pain (CPSP) [3], postoperative atrial fibrillation, acute lung injury [4], sepsis-associated organ dysfunction [5], fibrosis, thromboinflammation [6], and cancer progression.

In recent years, epigenetic regulation has emerged as a critical interface between perioperative stress and long-term pathophysiological consequences [7]. Epigenetic mechanisms modulate gene expression without altering the DNA sequence and include DNA methylation, histone acetylation, histone methylation, chromatin remodeling, and regulation by non-coding RNAs [8]. These mechanisms dynamically respond to environmental stimuli such as surgical trauma, ischemia–reperfusion injury, inflammation, hypoxia, anesthetic exposure, and metabolic stress [8]. Importantly, perioperative epigenetic reprogramming follows distinct temporal phases rather than representing a static process [9]. During the immediate perioperative period (minutes to hours), rapid and largely reversible changes in miRNA alterations, DNA methylation, histone modifications, and inflammatory signaling have been demonstrated in response to surgery and anesthesia [7,10]. These alterations may persist throughout the early postoperative phase (hours to days), where they contribute to immune regulation, tissue repair, and metabolic adaptation [11]. In contrast, more stable epigenetic remodeling (weeks to months), including persistent DNA methylation and histone modifications, has been described predominantly in experimental models and may contribute to long-term complications through mechanisms referred to as epigenetic memory. The time-dependent changes are shown in Table 1. However, the transition from adaptive perioperative stress responses to persistent epigenetic memory remains poorly defined in humans, and current evidence supports a temporal continuum rather than a distinct biological boundary.

Furthermore, epigenetic modifications are reversible, making them attractive therapeutic targets [16]. Growing evidence suggests that perioperative interventions including anesthetic techniques, regional anesthesia, local anesthetics, anti-inflammatory therapies, and metabolic modulation may influence postoperative outcomes through epigenetic pathways [14,17].

This review provides a comprehensive overview of perioperative epigenetics, focusing on neuroinflammation, NETosis, fibrosis, cardiac remodeling, immune dysfunction, and translational therapeutic approaches.

## 2. Fundamental Epigenetic Mechanisms in Perioperative Pathophysiology

### 2.1. DNA Methylation and Hydroxymethylation

DNA methylation typically occurs at CpG dinucleotides and is mediated by DNA methyltransferases (DNMTs) [18]. Hypermethylation of promoter regions usually suppresses gene transcription, whereas global hypomethylation may contribute to genomic instability and inflammatory activation.

Several perioperative studies have demonstrated altered DNA methylation patterns following surgery and anesthesia [7,19]. As an example, operative stress and surgical trauma seem to induce DNA methylation changes in immune cells. For instance, DNA hypomethylation of leucocytes has been associated with postoperative cognitive dysfunction [20]. In addition, anesthetics themselves seem to alter DNA methylation levels in various genes, as demonstrated for CHRNA7 following propofol administration [14], or to cause global changes. The various changes induced by anesthetics are reviewed by Elham Shahidi Delshad (2025) [15].

DNA methylation also plays important roles in cardiovascular disease, fibrosis, chronic pain, cancer biology and neuropathic pain, where persistent DNA methylation reprogramming has been identified in dorsal root ganglia after nerve injury.

**Figure 1:**
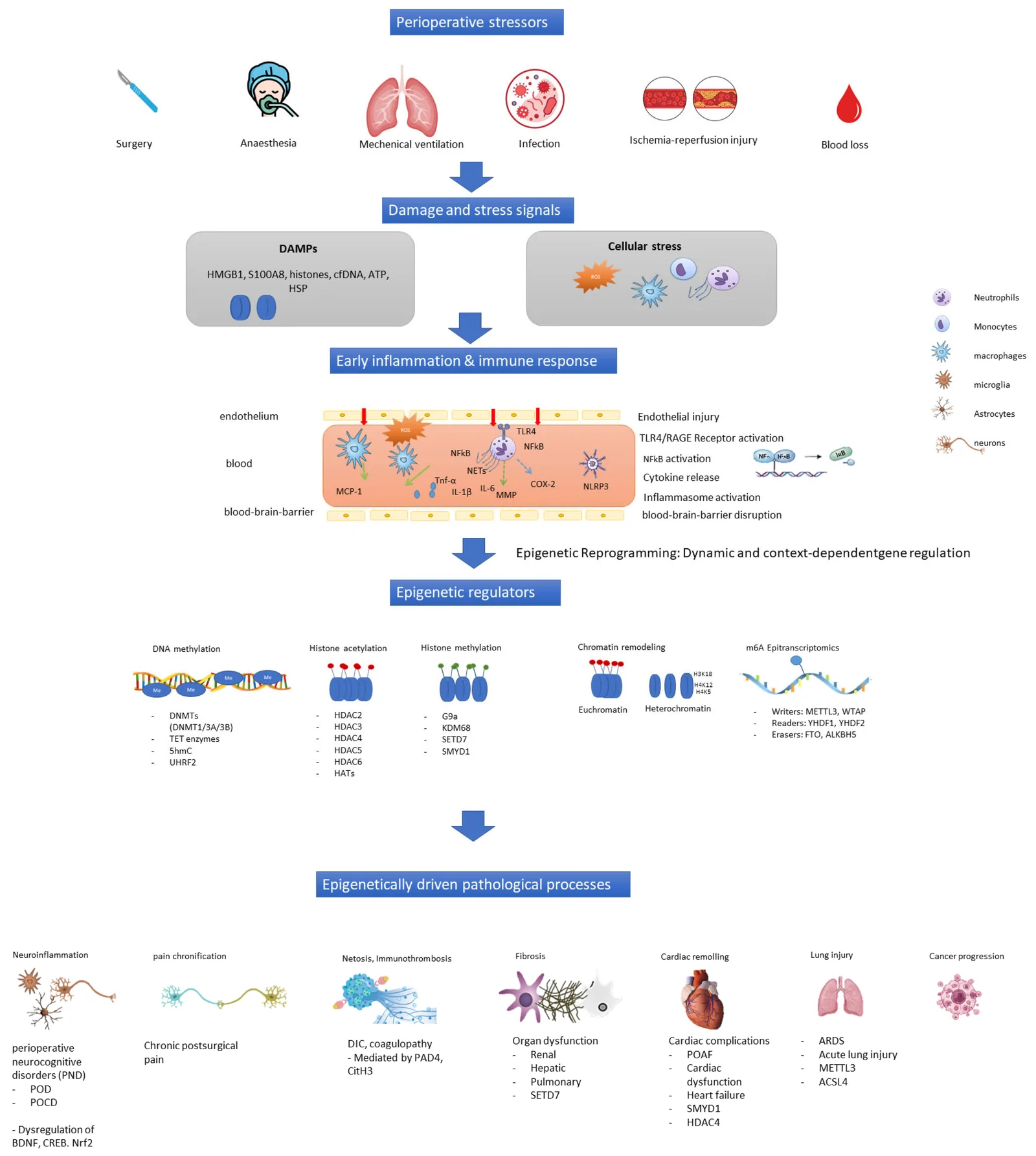
Integrated overview of epigenetic mechanisms linking perioperative stress to postoperative complications. Perioperative stressors, including surgery, anesthesia, mechanical ventilation, infection, ischemia–reperfusion injury, and blood loss, trigger the release of damage-associated molecular patterns (DAMPs), such as high-mobility group box 1 (HMGB1), S100A8, histones, circulating cell-free DNA (cfDNA), adenosine triphosphate (ATP), and heat shock proteins (HSPs), as well as cellular stress responses. These danger signals cross the endothelial barrier into the bloodstream (red arrows) and activate innate immune pathways through receptors such as toll-like receptor 4 (TLR4) and the receptor for advanced glycation end products (RAGE), resulting in nuclear factor kappa B (NF-κB) activation (the inset depicts IκB degradation and the subsequent release of NF-κB from the cytoplasmic NF-κB/IκB complex), inflammasome signaling, cytokine release, endothelial dysfunction, and blood–brain barrier disruption. The ensuing inflammatory response induces dynamic epigenetic reprogramming through multiple interconnected mechanisms, including DNA methylation, histone acetylation, histone methylation, chromatin remodeling, and N6-methyladenosine (m6A) RNA modification. Key epigenetic regulators include DNA methyltransferases (DNMTs), ten-eleven translocation (TET) enzymes, histone deacetylases (HDACs), histone acetyltransferases (HATs), histone methyltransferases and demethylases (e.g., G9a, SETD7, SMYD1, and KDM6B), as well as m6A writers (METTL3, WTAP), readers (YTHDF1/2), and erasers (FTO, ALKBH5). These epigenetic mechanisms regulate context-dependent gene expression and thereby influence inflammatory signaling, immune cell function, neuronal plasticity, tissue remodeling, and repair. Persistent dysregulation of these pathways contributes to epigenetically driven pathological processes, including perioperative neurocognitive disorders (PND), chronic postsurgical pain (CPSP), NETosis-associated immunothrombosis, fibrosis and multiple organ dysfunction, cardiac remodeling, acute lung injury/acute respiratory distress syndrome (ALI/ARDS), and cancer progression. The figure summarizes the central role of epigenetic reprogramming as the mechanistic link between perioperative stress, inflammation, and postoperative complications.

Hydroxymethylation, mediated by TET enzymes, has emerged as an additional layer of perioperative gene regulation. TET enzymes are a family of ten-eleven translocation (TET) methylcytosine dioxygenases. They are instrumental in DNA demethylation. TET-dependent DNA demethylation contributes to inflammatory regulation, fibrosis, and ischemia–reperfusion injury [21]. Altered hydroxymethylation (5hmC) is associated with POCD in animal models [22]. This effect seems to be mediated by suppression of the ubiquitin-like containing PHD and RING finger domains 2 (Uhrf2) [22], which protects genes from demethylation [23]. Recently a novel epigenetic mechanism wherein TET3 promotes Ectonucleotide Pyrophosphatase/Phosphodiesterase 1 (ENPP1) expression was described. TET3 acts through promoter hypomethylation and has emerged as a critical mediator of nonalcoholic fat liver disease (NAFLD). These findings provide new insight into the development of preventative measures for NAFLD [21]. Although these findings originate from non-perioperative disease models, they identify epigenetic pathways that may become relevant in perioperative organ injury and recovery (Figure 1).

### 2.2. Histone Acetylation and Histone Deacetylases

Histone acetylation generally promotes transcriptional activation by relaxing chromatin structure, whereas histone deacetylases (HDACs) suppress transcription through chromatin condensation [24].

HDAC signaling represents one of the most extensively studied epigenetic pathways in perioperative medicine. Multiple HDAC isoforms contribute to neuroinflammation [25,26], neuropathic pain, NET formation, fibrosis [27], and cardiac dysfunction.

Histone deacetylases (HDACs) comprise 18 isoforms that are classified into four classes according to sequence homology and cofactor dependency [28]. However, accumulating evidence indicates that individual HDAC isoforms exert highly tissue-specific functions in perioperative pathophysiology [29]. While HDAC2 and HDAC3 predominantly regulate neuroinflammation [30,31], synaptic plasticity and cognitive function, class II HDACs, including HDAC4 [32] HDAC5 and HDAC7 [33], contribute to cardiovascular remodeling and neuronal adaptation. In contrast, HDAC6 has emerged as an important regulator of cytoskeletal dynamics [34], immune cell activation and neutrophil extracellular trap (NET) formation [35].

As an example, class IIa HDACs elevate HMGB1-mediated inflammatory responses in the mouse brain [36], while HDAC3 might be a mediator of memory impairment and HDAC2 might contribute to neuropathic pain [37].

HDACs additionally regulate vascular and inflammatory pathways by influencing vascular homeostasis and thrombus resolution, highlighting a potential protective role of chromatin remodeling in cardiovascular disease [38,39] and might promote neutrophil extracellular trap (NET) formation [40] (Figure 1).

### 2.3. Histone Methylation and Chromatin Remodeling

Histone methylation exerts context-dependent transcriptional effects depending on the modified residue and degree of methylation. Histone methyltransferases such as G9a [41], SETD7 [42], and SMYD [42] proteins are critically involved in perioperative pathophysiology.

As an example, following nerve injury, the histone methyltransferase G9a is upregulated in primary sensory neurons, leading to H3K9-mediated transcriptional repression of multiple pain-regulating genes. This includes cannabinoid receptor 1 (CB1R), potassium channel genes, and μ-opioid receptors (MORs), resulting in neuronal hyperexcitability, impaired endogenous analgesic mechanisms, and reduced responsiveness to cannabinoid- and opioid-based therapies. Consequently, G9a-mediated epigenetic reprogramming contributes to the transition from acute to chronic neuropathic pain [41,43,44].

Chromatin remodeling also plays essential roles in cardiovascular disease. Chapski et al. demonstrated that adaptive chromatin remodeling precedes pathological phenotypes in heart failure and becomes reinforced during disease progression [45] (Figure 1).

### 2.4. m6A Epitranscriptomics and Emerging RNA-Based Epigenetic Regulation

N6-methyladenosine (m6A) represents the most abundant internal RNA modification in eukaryotic mRNA and regulates RNA stability, translation, splicing, and degradation. Recent studies suggest that m6A signaling might play major roles in fibrosis, sepsis-associated organ injury, neuroinflammation, neuronal injury, synaptic dysfunction and cancer biology. It also plays essential roles in neurodevelopment, synaptic plasticity, and memory formation, with its dysregulation linked to neurodegenerative conditions including Alzheimer’s and Parkinson’s diseases. Some evidence suggests that anesthetic agents and surgical stress can alter m6A methylation patterns in the hippocampus and other brain regions, potentially contributing to perioperative cognitive changes [9] (Figure 1).

These findings position epitranscriptomics as an emerging frontier within perioperative precision medicine.

## 3. Epigenetic Mechanisms of Perioperative Neurocognitive Disorders

Perioperative neurocognitive disorders (PNDs) encompass postoperative delirium [46], delayed neurocognitive recovery, and long-term postoperative cognitive dysfunction [47]. Aging, neuroinflammation, oxidative stress, blood–brain barrier disruption, and microglial activation are major contributors [48].

One of the main drivers of sterile perioperative inflammatory signaling is HMGB1 release. It promotes NF-κB activation, cytokine production, endothelial activation, microglial activation and neuroinflammation, which can aggravate cognitive dysfunction. There is evidence that NF-κB inhibition by Dexamethasone improves postoperative short-term cognitive impairment. This might be caused by reduced hippocampal neuroinflammation-mediated regulation of M1/M2 polarization, as well as inhibition of inflammatory HMGB1/RAGE/NF-κB signal transduction [49]. In addition, pretreatment with HMGB1 shRNA dramatically reduced both the degree of nuclear–cytoplasmic HMGB1 translocation of HMGB1 and NF-κB DNA binding in a mouse model of Alzheimer’s disease (AD). Together, the data indicate that HMGB1 mediates the pathogenesis of AD by activating RAGE/TLR4 signaling and that shRNA targeting HMGB1 may be a promising therapeutic strategy for treating AD [50], with potential applications to other cognitive diseases. Furthermore, the HMGB1 axis is closely related to TLR4 signaling. Additionally, inhibition of HMGB-1 expression level alleviates surgery-induced upregulation of toll-like receptor 4 (TLR4), nuclear factor-kappa B (NF-κB), and NLRP3 in the hippocampus of mice. Activation of the HMGB1/TLR4/NF-κB/NLRP3 axis contributes to postoperative neuroinflammation and cognitive impairment in murine models [51]. The HMGB1-TLR4 signaling further seems to link peripheral nerve injury to hippocampal neuroinflammation and cognitive dysfunction, highlighting a mechanistic connection between chronic pain and impaired cognition [52]. Chronic neuropathic pain is frequently accompanied by debilitating cognitive deficits; however, the further underlying mechanisms linking peripheral nerve injury to central nervous system dysfunction remain poorly understood, hindering the development of targeted therapies. Different anesthetic agents appear to converge on the HMGB1/NF-κB pathway through distinct upstream mechanisms. Dexmedetomidine primarily suppresses HMGB1/RAGE/NF-κB signaling [53], whereas volatile anesthetics such as sevoflurane have been associated with histone-dependent regulation of neuroinflammatory pathways and BDNF expression [54]. Together, these findings suggest that different anesthetic techniques may influence a common inflammatory–epigenetic signaling axis despite acting through different molecular targets.

As another important epigenetic mechanism, HDAC downregulation seems to amplify these inflammatory pathways.

One important contributor might be histone-binding protein RBBP4 (also known as RbAp48) [55], which is suspected to be the main cause of memory loss in normal aging. Perioperative chronic stress seems to exacerbate cognitive dysfunction after 6 h long-term isoflurane anesthesia. The activity of RbAp48/HDAC2-induced histone deacetylation modification plays a critical role in these negative effects on cognition [56]. HDAC-mediated inflammatory signaling additionally contributes to hippocampal dysfunction. Extracellular histones and chromatin fragments act as damage-associated molecular patterns (DAMPs) that activate TLR signaling, endothelial dysfunction, and coagulation cascades. Elevated circulating histone levels after cardiac surgery correlate with postoperative inflammation and adverse outcomes. These results suggest that surgery leads to PND through class IIa HDAC downregulation-triggered HMGB1 release in the hippocampus of aged mice. HDACs may be a potential therapeutic target for postoperative cognitive dysfunction [36]. Huang et al. demonstrated that downregulation of class IIa HDACs elevates HMGB1-mediated inflammatory responses in the brain of mice [36]. Zhang et al. identified HDAC3 as a key mediator of memory impairment after chronic constriction injury (CCI) [57], while Wang et al. showed that HDAC2-mediated dysregulation of glutamate transporters contributes to chemotherapy-induced neuropathic pain [37]. It is suggested that HDAC3 contributes to memory impairment after CCI by impairing synaptic plasticity in the hippocampus. HDAC3-dependent epigenetic remodeling contributes to hippocampal dysfunction and cognitive impairment following chronic pain states [57]. The loss of the balance between glutamate release and reuptake due to dysregulation of the solute carrier family 1 member 2 (SLC1A2; EAAT2)/vesicular glutamate transporter 2 (VGLUT2)/synaptophysin cascade in the spinal dorsal horn plays an important role in the development of paclitaxel-induced neuropathic pain. HDAC2-mediated repression of glutamate transporter pathways promotes central sensitization and neuropathic pain [37].

Histone trimethylation of H3K9 suppresses brain-derived neurotrophic factor (BDNF) expression and impairs hippocampal memory formation during anesthesia and surgery [58].

DNA methylation changes further contribute to PND. Laparotomy causes hippocampus-dependent cognitive decline by hypermethylating the NR2B gene, allowing us to understand the pathogenesis of PND in an epigenetic landscape [59]. Experimental evidence suggests that postoperative neurocognitive changes are accompanied by epigenetic alterations, particularly DNA methylation changes in genes involved in synaptic plasticity, learning, and memory. These epigenetic responses appear to be influenced by the pre-existing cognitive status and genetic background, including APOE genotype, indicating that individual susceptibility shapes the molecular response to surgical stress and anesthesia [60]. Li et al. reported that elderly patients with postoperative cognitive dysfunction exhibited global DNA hypomethylation of leucocyte DNA after hip surgery, suggesting systemic epigenetic dysregulation associated with neurocognitive decline [20]. Similarly, Zhong and Xu demonstrated altered DNA hydroxymethylation (5hmC) within the hypothalamus in aged mice with POCD [22]. They showed that loss of 5hmC in the hippocampus and the amygdaloid nucleus, modulated by ubiquitin-like containing PHD and RING finger domains 2 (Uhrf2) suppression, may result in the learning and memory ability impairment seen in mice with POCD [22]. UHRF2 mediates resistance to DNA methylation reprogramming in primordial germ cells [23]. Experimental evidence suggests that postoperative neurocognitive changes are accompanied by epigenetic alterations, particularly DNA methylation changes in genes involved in synaptic plasticity, learning, and memory. These epigenetic responses appear to be influenced by pre-existing cognitive status and genetic background, including the APOE genotype, indicating that individual susceptibility shapes the molecular response to surgical stress and anesthesia [22].

m6A-dependent pathways also contribute to anesthetic neurotoxicity and perioperative neurocognitive dysfunction.

m6A-dependent regulation of cellular metabolism influences microglial activation and neuroinflammatory responses associated with perioperative neurocognitive disorders [53]. m6A methylation also seems to be involved in the neuroprotective effects of hydrogen sulfide (H2S) against hippocampal neuronal apoptosis in aged rats with POCD. Experimental evidence suggests that restoration of m6A homeostasis may protect against neuronal injury and cognitive decline after surgery [61]. In addition, the m6A reader YTHDF1 improves sevoflurane-induced neuronal pyroptosis and cognitive dysfunction through augmenting CREB-BDNF signaling. YTHDF1 upregulation suppresses sevoflurane-induced activation of NLRP3 inflammation and pyroptosis in hippocampal tissues. YTHDF1-dependent m6A signaling promotes neuroprotective CREB/BDNF pathways and attenuates neuroinflammation-associated cognitive dysfunction [62].

Experimental evidence indicates that general anesthetics (like sevoflurane) contribute to perioperative neurocognitive disorders by inducing epigenetic remodeling, including alterations in histone methylation and chromatin regulation. These changes affect the expression of genes involved in synaptic plasticity and neuronal resilience, including synapse-associated proteins, the histone demethylase KDM5B [63], and the antioxidant transcription factor Nrf2 [64]. The resulting dysregulation of glutamatergic signaling, oxidative stress responses, and neuroinflammatory pathways promotes synaptic dysfunction and cognitive impairment. Together, these findings suggest that anesthesia itself, in addition to surgical stress, is an important driver of epigenetic mechanisms underlying postoperative neurocognitive dysfunction.

### Epigenetic Regulation of Chronic Postsurgical and Neuropathic Pain

Persistent postoperative pain is increasingly recognized as a neuroimmune disorder characterized by long-term epigenetic reprogramming within the peripheral and central nervous system.

Although primarily demonstrated in experimental models of nerve injury, these findings suggest that epigenetic remodeling may also contribute to the transition from acute postoperative pain to persistent postsurgical pain. Histone methyltransferase G9a (EHMT2)-dependent repression of genes encoding potassium channels and the cannabinoid receptor CNR1 (CB1R) promotes neuronal hyperexcitability and impairs endogenous analgesic pathways, supporting a broader role of chromatin remodeling in the chronification of postoperative pain [41].

Experimental evidence suggests that histone demethylation contributes to the development and maintenance of persistent pain by regulating neuroinflammatory gene expression. In particular, KDM6B-mediated demethylation of H3K27me3 enhances the transcription of pro-inflammatory genes, including IL6, through increased STAT3-dependent promoter activation. This epigenetic mechanism amplifies neuroinflammatory signaling within the dorsal root ganglia and spinal dorsal horn, thereby promoting pain sensitization and chronification [65].

Aberrant HDAC activity promotes neuropathic pain by driving epigenetic reprogramming of neuronal and immune cells, resulting in enhanced neuroinflammation, increased neuronal excitability, and persistent peripheral and central sensitization. These findings identify HDAC-dependent chromatin remodeling as a central mechanism in pain chronification and support the therapeutic potential of selective HDAC inhibitors [66].

Additional mechanisms include PARP-1-mediated TNF-α signaling, CXCR2 epigenetic regulation, suppression of μ-opioid receptor expression, and neuroimmune interactions involving microglia and astrocytes. Histone modification is an important epigenetic mechanism regulating incision-induced nociceptive sensitization. The spinal CXCR2 signaling pathway is one epigenetically regulated pathway controlling early and latent sensitization after incision [67]. Enhanced histone acetylation-mediated activation of CXCR2 signaling promotes spinal sensitization and contributes to the maintenance of chronic pain, highlighting chemokine pathways as important epigenetic mediators of nociceptive plasticity [68]. These results demonstrate key differences in the susceptibility of endogenous pain modulatory pathways and the adaptations of those pathways between male and female rats after TBI. These findings suggest that sex needs to be considered when designing mechanism-based therapies for pain after TBI [69].

These findings support the concept that chronic postoperative pain may represent an epigenetically maintained maladaptive neuroimmune state.

## 4. NETosis, Sterile Inflammation, and Immunothrombosis

Neutrophil extracellular traps are extracellular chromatin structures composed of DNA, histones, and granular proteins released by activated neutrophils. While NETs contribute to antimicrobial defense, excessive NET formation promotes thrombosis, endothelial injury, tissue damage, and organ dysfunction [70].

NETosis has emerged as a major mechanism linking perioperative inflammation, coagulation activation, and cancer progression [70]. Emerging evidence suggests that anesthetic techniques may differentially influence NETosis-associated inflammatory and chromatin-related pathways. In a prospective randomized study, Galoș et al. observed differences in postoperative NETosis- and angiogenesis-related biomarkers following intravenous and inhalational anesthesia, although intravenous lidocaine reduced NETosis-related marker expression irrespective of the anesthetic technique [71]. In contrast, Aghamelu et al. found no significant differences in serum NETosis expression between regional and volatile anesthesia, suggesting that NETosis may not represent a robust biomarker of anesthetic technique in breast cancer surgery [72].

An important enzyme in NET formation is peptidyl arginine deiminase 4 (PAD4) which converts arginine into citrulline. This conversion of positively charged arginine residues into neutral citrulline promotes chromatin decondensation. This process represents a crucial step in neutrophil extracellular trap (NET) formation, facilitating the release of decondensed chromatin decorated with antimicrobial proteins into the extracellular space. PAD4 is expressed in neutrophils that, when activated, can drive the formation of neutrophil extracellular traps. Hence, epigenetic regulation of PAD4 might alter NET formation. Experimental studies indicate that neutrophil extracellular traps (NETs) contribute to acute lung injury by disrupting endothelial barrier integrity and promoting inflammatory tissue damage. Histone citrullination, mediated by peptidylarginine deiminase 4 (PADI4), represents a key step in NET formation, and excessive release of citrullinated histone H3 has been shown to induce lung injury in experimental models. Pharmacological or genetic inhibition of PADI4 attenuates NET formation and reduces pulmonary damage, highlighting epigenetically regulated histone citrullination as a potential therapeutic target in acute inflammatory lung injury [73]. Boone et al. demonstrated in experimental pancreatic cancer models that NETs promote hypercoagulability through platelet activation and tissue factor release, whereas chloroquine reduces NET formation, platelet aggregation, circulating tissue factor levels, and hypercoagulability. Experimental studies demonstrated that NET-induced platelet activation depends on extracellular DNA and signaling through the receptor for advanced glycation end products (RAGE). Furthermore, PAD4-deficient mice, which are unable to generate NETs, exhibited reduced platelet aggregation and lower circulating tissue factor levels, highlighting the central role of PAD4-dependent NETosis in coagulation. Pharmacological inhibition of NET formation with chloroquine attenuated platelet aggregation, decreased tissue factor concentrations, and reduced hypercoagulability. Consistent with these findings, a randomized clinical study suggested that perioperative hydroxychloroquine treatment was associated with a lower incidence of venous thromboembolism (30% vs. 9.1%), although this reduction did not reach statistical significance [74]. Thus, while these data support the biological plausibility of NET inhibition as an antithrombotic strategy, they should be interpreted as hypothesis-generating rather than clinically practice-changing. The perioperative use of chloroquine or hydroxychloroquine for venous thromboembolism prevention requires validation in adequately powered, multicenter randomized controlled trials. In addition, potential safety concerns, including QT prolongation, drug interactions, retinal toxicity with prolonged exposure, hypoglycemia, and rare cardiomyopathy, must be carefully considered before clinical translation [75].

Experimental studies indicate that epigenetic regulation of histone acetylation plays a critical role in innate immune activation. HDAC-dependent histone deacetylation facilitates chromatin remodeling required for PADI4-mediated NET formation, thereby amplifying inflammatory responses and tissue injury. Pharmacological HDAC inhibition suppresses NETosis and mitigates experimental sepsis and acute lung injury, suggesting that modulation of histone acetylation may represent a promising therapeutic strategy for inflammatory perioperative complications [40].

NETs additionally contribute to acute lung injury, thrombosis, and postoperative complications. Surolia et al. demonstrated that NETosis participates in acute lung injury after chemical burns, while Boone et al. showed that chloroquine reduces cancer-associated hypercoagulability through NET inhibition in a murine pancreatic adenocarcinoma model. A dermal chemical burn by lewisite or phenylarsine oxide (PAO) resulted in Ca^2+^-associated peptidyl arginine deiminase 4 (PAD4) activation, NETosis, and ALI.

Experimental evidence indicates that neutrophil extracellular traps (NETs) contribute to thromboinflammation by promoting platelet activation, tissue factor release, and activation of the coagulation cascade. Pharmacological inhibition of NET formation attenuates hypercoagulability and thrombus formation in preclinical models, highlighting NET-targeted therapies as a promising strategy to reduce thromboembolic complications [75]. Overall, current evidence indicates that both intravenous and volatile anesthetics modulate inflammatory responses; however, direct evidence demonstrating differential epigenetic regulation of NETosis by specific anesthetic agents remains limited. Future studies integrating NET biomarkers with epigenetic readouts, including histone citrullination, histone acetylation, PAD4 activity, and DNA methylation, are required to better define these mechanisms.

## 5. Cardiac Epigenetics and Perioperative Cardiovascular Remodeling

Cardiovascular disease is increasingly recognized as an epigenetically regulated disorder involving DNA methylation, chromatin remodeling, histone modification, metabolic signaling, and inflammatory activation.

DNA methylation patterns seem to predict susceptibility to cardiac pathology and postoperative complications. Fischer et al. identified DNA methylation signatures associated with postoperative atrial fibrillation after cardiac surgery [10].

Accumulating evidence suggests that DNA methylation contributes to cardiac remodeling and heart failure. In this context, Chen et al. demonstrated that DNA methylation patterns determine susceptibility to isoproterenol-induced cardiac pathology and correlate with distinct chromatin states. Using the first single-base resolution map of the mammalian cardiac DNA methylome together with a case–control analysis of heart failure, the authors identified widespread disease-associated alterations in DNA methylation, supporting an important role for epigenetic regulation in cardiac disease progression [76].

Adaptive chromatin remodeling occurs early during cardiac stress responses and precedes overt pathological remodeling. Cardiac stress induces extensive chromatin remodeling that precedes overt structural and functional deterioration of the myocardium. These epigenetic alterations emerge during the initial compensatory phase and persist throughout disease progression, thereby promoting maladaptive gene expression programs associated with cardiac hypertrophy, fibrosis, inflammation, and ventricular dysfunction. Chapski et al. demonstrated that progressive reorganization of chromatin architecture reinforces pathological transcriptional states during the transition from compensated remodeling to overt heart failure. These findings suggest that persistent epigenetic reprogramming contributes to the maintenance and progression of heart failure and may represent a potential target for therapeutic intervention [45].

Histone-modifying enzymes also regulate endothelial function and fibrinolysis. HDAC inhibition increases endothelial tissue plasminogen activator release and may improve vascular homeostasis.

Emerging evidence suggests that histone lactylation links metabolic reprogramming to epigenetic regulation during sepsis. Experimental studies demonstrate that lactate-induced histone lactylation can modulate the expression of epigenetic regulators, including METTL3, thereby promoting m6A RNA methylation of ferroptosis-related genes such as ACSL4. This epigenetic crosstalk enhances ferroptotic cell death and contributes to sepsis-associated acute lung injury, highlighting the interplay between metabolic, chromatin, and RNA modifications in inflammatory organ dysfunction [77]. Additional studies have identified m6A-related crosstalk with DNA methylation and non-coding RNAs in cardiac and liver fibrosis. In cardiac fibrosis dysregulated mitochondrial fission drives pathological cellular states, and its upstream regulatory mechanisms may be driven by epitranscriptomic regulation, particularly N6-methyladenosine (m6A) modification. Recent findings indicate the involvement of an epitranscriptomic pathway comprising METTL3 and YTH N6-methyladenosine RNA binding protein F2 (YTHDF2), which regulates PGC-1α through m6A modification. m6A-dependent regulation of mitochondrial dynamics contributes to fibroblast activation and progression of cardiac fibrosis [78].

Emerging evidence indicates that epigenetic histone modifications contribute to cardiac responses to physiological and pathological stress. Muscle-specific histone methyltransferases, including SMYD1, regulate transcriptional networks controlling cardiomyocyte growth, differentiation, and remodeling. Disruption of these chromatin-dependent regulatory mechanisms promotes pathological hypertrophy and heart failure, suggesting that modulation of histone methylation may represent a promising therapeutic strategy for preventing stress-induced cardiac dysfunction [79].

Circulating histones have emerged as potential biomarkers of cardiac surgery-associated inflammation and endothelial injury. Gao et al. demonstrated that elevated histone levels after cardiac surgery correlate with postoperative prognosis [80]. Circulating histones reached peak levels faster than NT-proBNP, PCT and CRP. Furthermore, peak histone levels correlated with biomarkers and postoperative clinical outcomes. Circulating histones may therefore be used as a prognostic indicator for patients after cardiac surgery with cardiopulmonary bypass (CPB) [80].

Perioperative ischemia–reperfusion injury induces complex molecular responses involving oxidative stress, mitochondrial dysfunction, apoptosis, and inflammatory signaling, processes that are increasingly recognized to be regulated by epigenetic mechanisms. Experimental evidence demonstrates that pharmacological modulation of apoptosis, for example by lidocaine, reduces cardiomyocyte death and limits myocardial injury, highlighting the therapeutic potential of targeting stress-responsive signaling pathways [81].

## 6. Fibrosis, Organ Dysfunction, and Metabolic–Epigenetic Crosstalk

Fibrosis is a common endpoint of chronic inflammation and tissue injury. Epigenetic mechanisms critically regulate fibroblast activation, extracellular matrix production, epithelial-to-mesenchymal transition (EMT), and myofibroblast differentiation.

Recent studies highlight extensive crosstalk between metabolism and chromatin regulation in fibrosis. Histone acetylation, methylation, and m6A RNA modifications dynamically regulate fibrotic gene programs.

Emerging evidence indicates that histone methylation is a key epigenetic regulator of renal fibrogenesis. Histone methyltransferases such as SETD7 (SET7/9) promote inflammatory signaling, macrophage polarization, myofibroblast activation, and extracellular matrix deposition through H3K4 methylation and activation of profibrotic pathways, including NF-κB and TGF-β signaling. Pharmacological inhibition of SETD7 attenuates inflammation, suppresses epithelial-to-mesenchymal transition and myofibroblast differentiation, and reduces renal fibrosis in experimental models, highlighting histone methylation as a promising therapeutic target for chronic kidney injury [82].

Accumulating evidence indicates that m6A RNA methylation is an important regulator of liver fibrosis. Epitranscriptomic modifiers, including the WTAP-associated m6A methylation complex, together with RNA-binding proteins such as TTP, control the expression of inflammatory and profibrotic mediators that govern hepatic stellate cell activation and fibrotic remodeling. Targeting these pathways may therefore represent a novel therapeutic approach for chronic liver injury [83].

Emerging evidence additionally implicates epigenetic regulation in ischemia–reperfusion injury, acute kidney injury, and acute lung injury.

NETosis, HMGB1 signaling, oxidative stress, and mitochondrial dysfunction represent central converging pathways linking perioperative inflammation and organ fibrosis.

## 7. Translational and Therapeutic Perspectives

Because epigenetic modifications are potentially reversible, epigenetic therapies represent attractive candidates for perioperative precision medicine.

Potential therapeutic approaches include:

• HDAC inhibitors;
• DNMT inhibitors;
• BET bromodomain inhibitors;
• PAD4 inhibitors;
• Histone methyltransferase inhibitors;
• m6A-targeting therapies;
• Anti-NETosis strategies;
• Metabolic–epigenetic modulators.

From a therapeutic perspective, the efficacy of HDAC inhibition appears to depend on isoform selectivity. Broad-spectrum HDAC inhibitors such as trichostatin A or vorinostat demonstrate anti-inflammatory effects in several experimental models but may also induce off-target effects due to their limited specificity. Consequently, recent research has increasingly focused on selective HDAC inhibitors targeting individual isoforms, particularly HDAC3 and HDAC6, which have shown promising results in experimental models of perioperative neurocognitive disorders, acute lung injury, and NETosis while potentially reducing unwanted systemic effects. Nevertheless, clinical evidence for selective HDAC inhibition in perioperative medicine remains scarce.

Histone deacetylase (HDAC) inhibitors represent one of the most advanced classes of epigenetic therapeutics. However, their stage of clinical development differs substantially between compounds. Broad-spectrum (pan-) HDAC inhibitors such as vorinostat, romidepsin, belinostat, and panobinostat [84] are already approved for selected hematological malignancies, demonstrating the clinical feasibility of pharmacological HDAC inhibition. In contrast, selective HDAC inhibitors targeting individual isoforms, including HDAC3- and HDAC6-selective compounds, remain largely in preclinical or early translational development [85,86]. Experimental studies have demonstrated beneficial effects of selective HDAC inhibition in models of postoperative neurocognitive disorders, acute lung injury, ARDS, neuropathic pain, and NETosis. Nevertheless, none of these compounds has yet been evaluated in adequately powered clinical trials for perioperative indications. Therefore, translation into perioperative medicine requires further studies addressing efficacy, safety, optimal timing, and isoform specificity.

Single-cell epigenomics, multi-omics integration, and liquid biopsy technologies may enable individualized perioperative risk stratification and biomarker-guided therapies.

Future studies should focus on:

• Identification of perioperative epigenetic biomarkers;
• Longitudinal characterization of epigenetic trajectories;
• Integration of transcriptomic and epigenomic data;
• Organ-specific epigenetic signatures;
• Precision anesthetic strategies targeting epigenetic pathways.

Importantly, accumulating evidence suggests that commonly used anesthetic agents can directly modulate epigenetic pathways relevant to perioperative outcomes. In an in vitro model, propofol induced DNA methylation changes in genes involved in cholinergic neurotransmission, highlighting a potential mechanism through which anesthetic exposure may affect postoperative neurocognitive function [9]. Similarly, midazolam was shown to alter the methylation status of acetyl- and butyrylcholinesterase genes, providing a possible epigenetic link between perioperative sedation and postoperative delirium susceptibility [10]. These findings support the concept that anesthetic agents not only modulate neuronal activity acutely but may also induce persistent epigenetic changes affecting neuroinflammatory and cognitive pathways. The clinical relevance of perioperative neuroinflammation is further supported by the CONFUSED study, which identified associations between inflammatory signaling and the development of postoperative delirium in critically ill and surgical patients [87]. Together with emerging evidence on anesthetic-induced epigenetic regulation, these findings suggest that neuroinflammatory and epigenetic pathways may converge in the pathogenesis of perioperative neurocognitive disorders and could serve as future targets for biomarker-guided risk stratification and personalized perioperative interventions. Although the CONFUSED study did not directly investigate epigenetic mechanisms yet, its findings support the concept that perioperative inflammatory responses may represent upstream triggers of epigenetic reprogramming in vulnerable patients.

### Challenges and Future Perspectives

Despite the rapidly growing body of evidence highlighting the importance of epigenetic mechanisms in perioperative medicine, several challenges currently limit their translation into clinical practice. First, the available literature is characterized by considerable heterogeneity. Differences in experimental models, anesthetic protocols, surgical procedures, patient populations, sampling time points, and the epigenetic markers investigated make direct comparison between studies difficult. Furthermore, most studies focus on individual epigenetic mechanisms, although DNA methylation, histone modifications, RNA modifications, and non-coding RNAs are highly interconnected and act within complex regulatory networks. Consequently, conflicting findings across studies likely reflect methodological differences rather than true biological contradictions.

A second major challenge is the translation of promising preclinical findings into clinical applications. Most mechanistic evidence has been obtained from animal models or in vitro studies, whereas prospective longitudinal human studies remain scarce. Although several epigenetic targets, including HDACs, PAD4, DNMTs, and m6A-modifying enzymes, have demonstrated therapeutic potential in experimental models of perioperative neurocognitive disorders, acute lung injury, fibrosis, and NETosis, robust clinical evidence is still lacking. In addition, tissue-specific epigenetic regulation, interindividual variability, and potential off-target effects complicate the clinical development of epigenetic therapies. Future multicenter prospective studies and randomized controlled trials will therefore be essential to establish efficacy, safety, and optimal patient selection.

Another important consideration is the temporal nature of perioperative epigenetic regulation. Current evidence suggests that epigenetic remodeling represents a continuum ranging from rapid and largely reversible molecular adaptations during surgery and anesthesia to more persistent alterations that may contribute to long-term postoperative complications. However, the transition from acute adaptive stress responses to stable epigenetic memory remains poorly understood, particularly in humans. Defining these temporal dynamics through longitudinal sampling before surgery, throughout the postoperative period, and during long-term follow-up will be critical for identifying optimal therapeutic windows and developing biomarker-guided precision medicine approaches.

Ultimately, future research should integrate multi-omics technologies, longitudinal clinical cohorts, and functional experimental models to better define causal epigenetic mechanisms and accelerate the translation of epigenetic discoveries into personalized perioperative care.

## 8. Conclusions

Epigenetic mechanisms have emerged as central regulators of perioperative pathophysiology, linking surgical trauma, anesthesia, inflammation, ischemia–reperfusion injury, and metabolic stress to persistent alterations in gene expression and cellular function. DNA methylation, histone modifications, chromatin remodeling, non-coding RNAs, and m6A-mediated epitranscriptomic regulation collectively orchestrate neuroinflammatory responses, immune dysfunction, NETosis, fibrosis, cardiovascular remodeling, and organ injury across the perioperative period.

A growing body of evidence indicates that epigenetic reprogramming contributes to the development of perioperative neurocognitive disorders, chronic postsurgical pain, immunothrombosis, and postoperative organ dysfunction by sustaining maladaptive inflammatory and stress-response pathways. Key regulatory nodes, including HDACs, G9a, HMGB1/NF-κB signaling, NETosis-associated chromatin pathways, and m6A regulators such as METTL3 and YTHDF proteins, have emerged as important mediators of these processes and represent promising therapeutic targets.

Future research in perioperative epigenetics should focus on three complementary directions. First, the identification and validation of robust epigenetic biomarkers, including DNA methylation signatures, histone modifications, circulating cell-free DNA, and RNA-based biomarkers, will be essential for early diagnosis, risk stratification, and prediction of postoperative complications. Second, promising preclinical findings on epigenetic therapeutics, particularly HDAC, PAD4, DNMT, and m6A-targeting agents, should be translated into well-designed clinical trials to evaluate their efficacy, safety, optimal timing, and patient selection in the perioperative setting. Third, increasing evidence suggests that anesthetic agents themselves modulate epigenetic pathways, highlighting the need for individualized anesthesia strategies that integrate patient-specific epigenetic profiles with perioperative management. Such precision medicine approaches may ultimately enable personalized prevention and treatment of postoperative complications while improving long-term patient outcomes.

Importantly, the reversible nature of epigenetic modifications provides unique opportunities for therapeutic intervention. Advances in single-cell epigenomics, liquid biopsy technologies, and multi-omics integration are expected to facilitate the identification of perioperative biomarkers and enable individualized risk stratification. In the future, the integration of epigenetic profiling into perioperative care may support precision medicine approaches that improve patient outcomes through targeted prevention and treatment of postoperative complications.

Taken together, epigenetic regulation represents a unifying framework that links perioperative stress responses to long-term clinical outcomes. Further translational and clinical studies are required to validate epigenetic biomarkers and to determine whether targeted modulation of epigenetic pathways can be successfully translated into perioperative precision therapeutics. 

## Figures and Tables

**Table 1:** Time dependency of perioperative epigenetic changes.

Time	Dominating Epigenetic Changes	Biological Consequence	References
Minutes to hours	Histone acetylation, histone methylation, chromatin remodeling, miRNAs	Acute stress response, cytokine expression	[7,11,12,13]
Hours to days	ncRNAs, DNA methylation, histone methylation	Inflammation, tissue repair, delirium	[11,14]
Weeks	Persisting chromatin remodeling, DNA methylation	Recovery or long-term dysfunction	[15]
Month to years	Stable DNA methylation, epigenetic memory	Chronical inflammation chronical pain, postoperative cognitive dysfunction	[15]

## Data Availability

No new data were created or analyzed in this study. Data sharing is not applicable to this article.
